# Thrombus aspiration in primary percutaneous coronary intervention in acute ST-elevation myocardial infarction patients with high thrombus burden: one-year outcomes in a tertiary healthcare center in Ho Chi Minh City

**DOI:** 10.1186/s43044-024-00583-2

**Published:** 2024-12-01

**Authors:** Duy Cao Phuong Le, Giang Thai Pham, Quan Duy Vo

**Affiliations:** 1 Department of Cardiovascular Intervention, Nguyen Tri Phuong Hospital, Ho Chi Minh City, Vietnam; 2 Military Central Hospital 108, Ha Noi, Vietnam; 3 Faculty of Medicine, Nguyen Tat Thanh University, Ho Chi Minh City, Vietnam

**Keywords:** Percutaneous coronary intervention, Thrombus aspiration, Major adverse cardiovascular events, STEMI, Thrombus burden

## Abstract

**Background:**

Primary percutaneous coronary intervention (PCI) can dislodge atherosclerotic debris, risking microvascular embolism. Thrombus aspiration (TA) before stenting in ST-segment elevation myocardial infarction (STEMI) patients has been linked to reduced mortality, lower recurrence of heart attacks, and improved cardiac function. However, limited research exists on the effectiveness of TA in Vietnam, underscoring the need for further studies to enhance cardiovascular care. This prospective observational study was conducted to evaluate the role of TA in STEMI patients admitted with a substantial thrombus burden at Nguyen Tri Phuong Hospital.

**Results:**

Out of 92 participants, 68 underwent TA treatment. Post-treatment, the TA group exhibited better TIMI and TMP flow grades and a higher rate of ST-segment normalization, with no significant difference in major adverse cardiac events (MACEs) at 30-day and 12-month follow-ups compared to those untreated.

**Conclusions:**

TA during PCI enhances ST-segment normalization and TIMI and TMP scores in STEMI patients, improving myocardial perfusion. No difference in MACE occurrence was noted between groups after 30 days and 12 months, suggesting TA’s potential benefits without increasing adverse outcomes.

## Background

Cardiovascular diseases represent the foremost causes of mortality and morbidity worldwide, accounting for an estimated 18 million deaths annually [1]. Coronary artery disease, particularly acute myocardial infarction (AMI), is among the top causes of death, even with significant advancements in treatment. AMI constitutes a severe medical emergency marked by the necrosis of heart muscle tissue due to an insufficient blood supply [2]. The implementation of primary percutaneous coronary intervention (PCI) in the management of acute ST-elevation myocardial infarction (STEMI) has significantly enhanced patient survival rates and mitigated the severity of the disease [3]. However, even after successful revascularization of the main coronary artery, myocardial blood flow may remain inadequate due to persistent microvascular obstruction and dysfunction [4].

Thrombus aspiration (TA) has been introduced as an adjunctive technique during revascularization to improve microvascular perfusion and facilitate blood flow to the myocardium, especially in patients with a considerable thrombus load. Although theoretically beneficial, randomized clinical trials have produced inconsistent evidence regarding its efficacy [5, 6]. Given these discrepancies, our study aims to evaluate the efficacy of TA in conjunction with primary PCI for STEMI patients with a significant thrombus burden, particularly within the context of limited resources in low- and middle-income countries such as Vietnam.

## Methods

### Design of the study

This prospective observational study was conducted at Nguyen Tri Phuong Hospital in Ho Chi Minh, Vietnam. Each participant was provided written informed consent prior to enrollment. The study’s protocol received approval from the Nguyen Tri Phuong Hospital Ethical Board.

### Study population

**Fig. 1:**
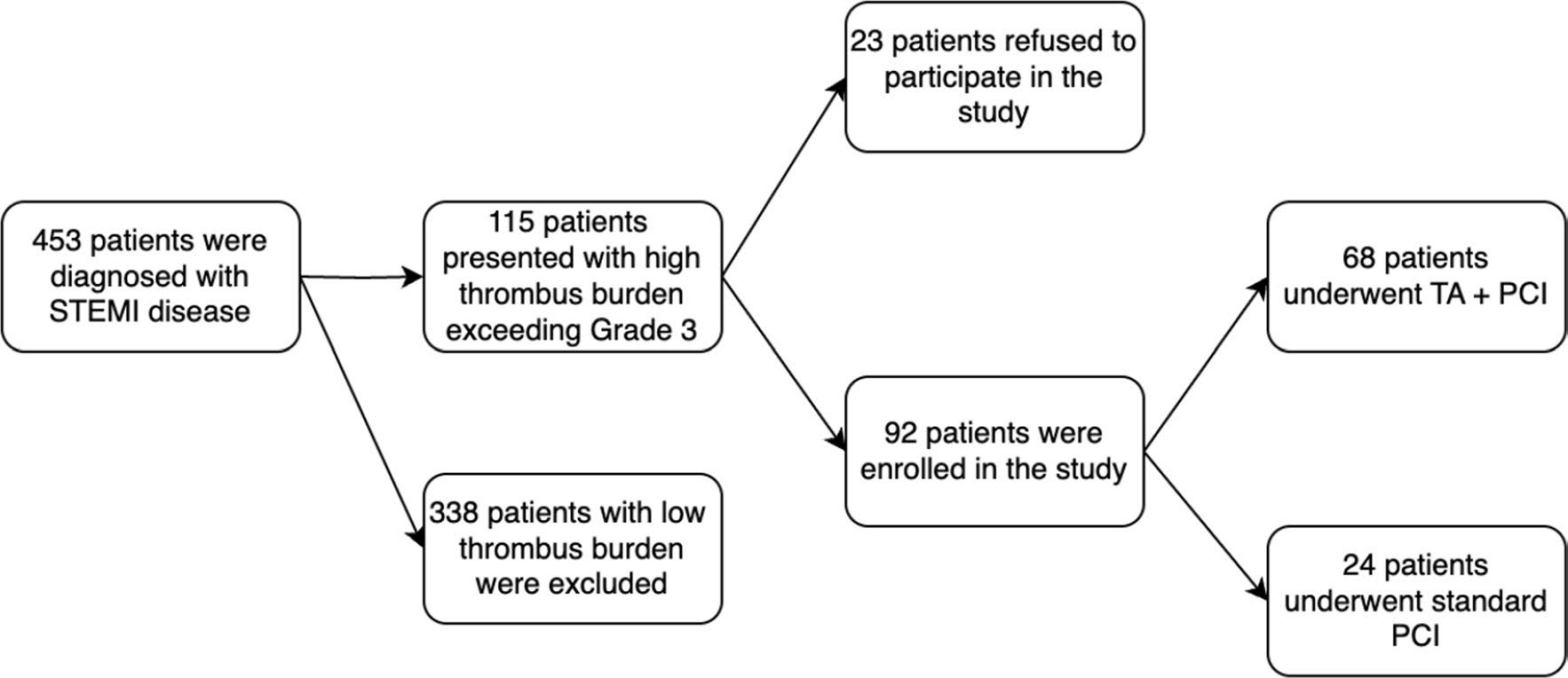
Study flowchart

The study enrolled 92 adult patients with STEMI who underwent PCI and presented a significant thrombus burden on coronary angiography. Of these patients, 68 consented to TA before stenting, while the remaining 24 received conventional PCI without TA. Patients with a low thrombus burden were excluded from the study (Fig. 1).

All participants underwent a comprehensive evaluation, including a detailed medical history, thorough clinical examination, laboratory testing, cardiac biomarker assessment, and echocardiography upon admission. AMI was defined according to the Fourth Universal Definition of Myocardial Infarction [7]. The diagnosis of STEMI was made upon the presence of chest pain suggestive of myocardial ischemia lasting more than 30 min, accompanied by ST-segment elevation in two or more contiguous leads or a new left bundle branch block on the ECG.

The assessment of thrombus burden through angiographic evaluation was categorized as follows: Grade 0 indicated the absence of a thrombus; Grade 1 suggested a possible presence of a thrombus; Grade 2 was characterized by the largest dimension of the thrombus being less than half the vessel’s diameter; Grade 3 was defined when the largest dimension of the thrombus was more than half but less than two times the vessel’s diameter; Grade 4 was determined when the largest dimension of the thrombus exceeded two-vessel diameters; and Grade 5 was identified in cases of complete vessel occlusion due to a thrombus [8]. The thrombus aspiration procedure was indicated only for patients with a thrombus burden exceeding Grade 3 (G3).

### Catheterization procedure

Patients presenting with STEMI symptoms within 12 h were considered for PCI according to the European Society of Cardiology (ESC) guidelines for STEMI management [9]. All suspected STEMI cases received an initial 12-lead ECG within 10 min of arrival in the Emergency Department. Patients were administered loading doses of aspirin (300–400 mg) and an ADP receptor inhibitor (600 mg of clopidogrel or 180 mg of ticagrelor). Subsequently, they were immediately transferred to the cardiac catheterization laboratory. During the procedure, intravenous unfractionated heparin (UFH) therapy was administered. Coronary angiography was carried out via the radial or femoral artery to identify and cross the culprit lesion with an angioplasty guidewire. After PCI, patients were transferred to the post-procedure division of the Cardiovascular Intervention Department and subsequently discharged if they maintained stability for 24 h. Post-procedure anticoagulation therapy with UFH or low molecular weight heparin (LMWH) was determined at the operator’s discretion.

### *Thrombus* aspiration procedure

Following the initial diagnostic coronary angiography, the culprit lesion was identified. Manual thrombus aspiration was then carried out using the Diver CE (Invatec, Brescia, Italy) at the discretion of the operating physician. The Diver CE is designed for rapid exchange and is compatible with 6-F catheters. It features a central aspiration lumen (0.6604 mm) and is equipped with a soft, flexible, non-traumatic tip with multiple holes connected to the central lumen. Aspiration is executed by attaching a 20-mL syringe to the catheter. The aspiration process would be terminated upon the successful extraction of thrombotic material and when subsequent attempts resulted in no debris or if, after several unsuccessful attempts, no visible material was extracted. Following manual TA, stent implantation and balloon dilation (both pre-dilation and post-dilation) were considered to enhance procedural outcomes as deemed necessary. The decision to perform manual TA, the choice and quantity of stents, the employment of additional devices, and the application of angiographic selection criteria, such as the detection of visible thrombus on angiography, were all subject to the operator’s judgment. These interventions were followed by conventional PCI targeting the culprit vessel.

### Follow-up

After the procedure, patients without any contraindications were prescribed aspirin (81 mg daily) and either clopidogrel (75 mg daily) or ticagrelor (90 mg twice daily) for a minimum duration of 12 months. Additional medications, including statins, beta-blockers, angiotensin-converting enzyme inhibitors (ACEIs), angiotensin receptor blockers (ARBs), and spironolactone, were administered in alignment with ESC guidelines [9]. All patients were enrolled in an outpatient follow-up program within our department. According to the hospital’s protocol, follow-up visits were made 7 and 30 days after discharge, followed by monthly check-ups.

### Outcome measures

Data were collected on baseline clinical characteristics, coronary artery lesion features observed on angiograms, and specific details of the PCI procedures. Coronary lesions were classified following the American College of Cardiology/American Heart Association (ACC/AHA) classification system into three groups—A, B, and C [10].

The primary outcomes we assessed in our study included major adverse cardiac events (MACE) such as all-cause mortality, stent thrombosis (ST), myocardial infarction (MI), target vessel revascularization (TVR), and stroke, evaluated at 30 days and 12 months post-procedure. Additional outcomes were also assessed, including relief from chest pain, improvement in TIMI and TMP scores, and the overall success of the procedure.

Optimal reperfusion post-intervention was identified by normal ST-segment recovery and thrombolysis in myocardial infarction (TIMI) flow grade of 3. Successful TA was indicated by macroscopic thrombus visible in the filter and an enhancement in coronary flow, as reflected by an improved TIMI score post-TA.

A technically successful coronary intervention was defined by no stenosis greater than 20%, restoration of normal coronary flow (TIMI = 3), and the absence of distal embolization. Clinical success in coronary intervention was defined as technical success accompanied by an absence of any MACE during the initial hospital stay.

ST-segment recovery was classified into three categories: normal recovery, where the ST-segment showed improvement of ≥ 70% compared to the baseline; partial improvement, with ST-segment recovery between 30–70% relative to baseline; and no change, where ST-segment recovery was < 30% compared to baseline. Stent thrombosis was categorized according to the Academic Research Consortium definitions into definite, probable, or possible [11].

### Statistical analysis

Data from the study were analyzed using SPSS v20 (IBM, USA). For quantitative variables, we calculated the mean and standard deviation for normally distributed data, employing the t test to assess differences between the two groups. For variables not following a normal distribution, we described them using the median and interquartile range and utilized the Wilcoxon rank-sum test to evaluate differences between groups.

Qualitative variables were summarized using frequencies and percentages. To compare the characteristics of these variables between groups, we applied the chi-square test or Fisher’s exact test, depending on the appropriateness of the data distribution and sample size. A p value of less than 0.05 was considered indicative of statistical significance.

## Results

### Patient demographics

Our study included 92 patients with an average age of 62.4 years. Most patients were male (78.3%), with similar proportions in both groups (*p* = 0.65). BMI, hypertension, diabetes, and smoking status were also comparable between the groups, with no significant differences observed (all *p* > 0.38) (Table 1).

**Table 1:** Patient demographics

Characteristic	Total (*n* = 92)	With TA (*n* = 68)	Without TA (*n* = 24)	*p* value
Age (Mean ± SD)	62.4 ± 11.8	63.1 ± 11.6	60.2 ± 12.3	0.38
Male gender	72 (78.3%)	54 (79.4%)	18 (75.0%)	0.65
BMI (kg/m^2^) (Mean ± SD)	25.6 ± 3.4	25.8 ± 3.5	25.1 ± 3.3	0.49
Hypertension	52 (56.5%)	38 (55.9%)	14 (58.3%)	0.83
Diabetes mellitus	28 (30.4%)	21 (30.9%)	7 (29.2%)	0.87
*Smoking status* Current smoker Former smoker Non-smoker	34 (37.0%) 28 (30.4%) 30 (32.6%)	25 (36.8%) 20 (29.4%) 23 (33.8%)	9 (37.5%) 8 (33.3%) 7 (29.2%)	0.95 0.72 0.68
Chronic kidney disease	10 (10.9%)	7 (10.3%)	3 (12.5%)	0.78

Table 2 summarizes the procedural characteristics of patients with and without TA. All patients received aspirin, while 70.7% also received clopidogrel and 29.3% ticagrelor. Anticoagulation was mostly achieved with unfractionated heparin (82.6%). Disease-type distribution was primarily one-vessel (59.8%) and two-vessel (30.4%), with similar lesion locations and ACC/AHA classifications between groups. Post-procedure anticoagulation therapy was used in 29.3% of patients, with no significant differences observed across groups.

**Table 2:** Procedural characteristics

Characteristic	Total (n = 92)	With TA (n = 68)	Without TA (n = 24)	*p* value
*Pre-procedure anti platelet therapy*				
Aspirin	92 (100%)	68 (100%)	24 (100%)	0.98
Clopidogrel	65 (70.7%)	48 (70.6%)	17 (70.8%)	
Ticagrelor	27 (29.3%)	20 (29.4%)	7 (29.2%)	
*Pre-procedure anticoagulant therapy*				
UFH	76 (82.6%)	56 (82.4%)	20 (83.3%)	0.82
LMWH	16 (17.4%)	12 (17.6%)	4 (16.7%)	
*Type of disease*				
one-vessel	55 (59.8%)	40 (58.8%)	15 (62.5%)	0.8
two-vessel	28 (30.4%)	21 (30.9%)	7 (29.2%)	
three-vessel	9 (9.8%)	7 (10.3%)	2 (8.3%)	
*Location of lesion*				
RCA	32 (34.8%)	23 (33.8%)	9 (37.5%)	0.7
LAD	45 (48.9%)	34 (50.0%)	11 (45.8%)	
LCx	15 (16.3%)	11 (16.2%)	4 (16.7%)	
*ACC/AHA coronary lesions classification*				
A	22 (23.9%)	16 (23.5%)	6 (25.0%)	0.8
B	45 (48.9%)	33 (48.5%)	12 (50.0%)	
C	25 (27.2%)	19 (27.9%)	6 (25.0%)	
Post-procedure anticoagulant therapy	27 (29.3%)	19 (28%)	8 (33.4%)	0.3

### TA results and factors related to successful TA

**Fig. 2:**
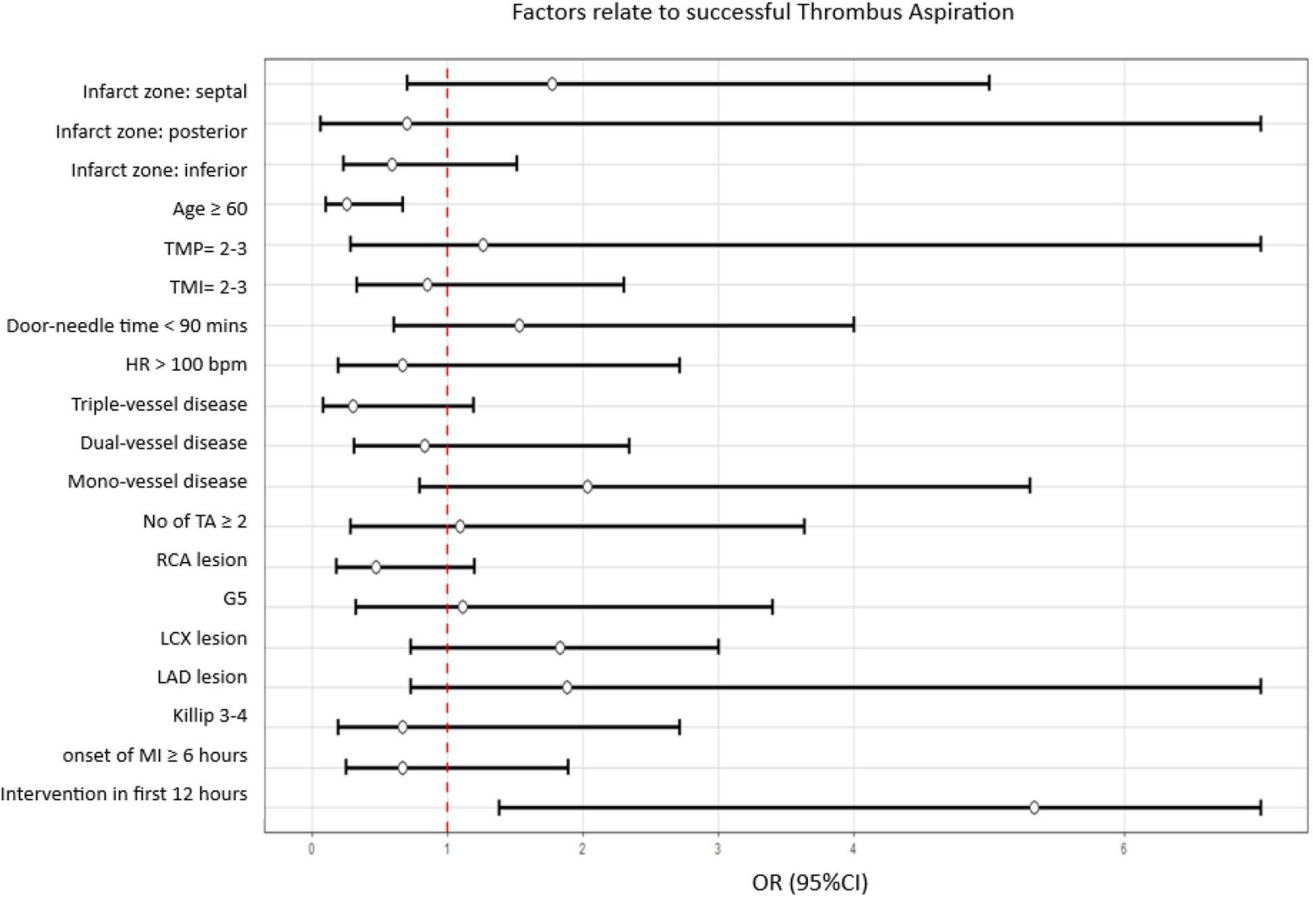
Factors related to successful TA

In our study, TA procedure was employed in 68 patients (73.9%) during PCI, while 24 patients denied this procedure. The baseline characteristics of the study participants are outlined in Table S1. Success aspiration was reported in 54 patients (79.4%) who underwent TA, with no post-procedural complications noted. The predominant reason for the unsuccessful TA was the absence of visible thrombus debris (Table S2). Additionally, multivariate analysis revealed that the probability of TA success diminished for patients older than 60 years. In contrast, initiating the intervention within the first 12 h significantly improved the chances of successful TA (Fig. 2).

### Major adverse cardiovascular events after 12 months following thrombus aspiration during primary PCI in STEMI patients

All participants in the study showed significant enhancements in both TIMI and TMP scores after PCI (Table 2). However, patients receiving TA treatment experienced a higher rate of ST-segment normalization (86.8% versus 26.1%, *p* < 0.001), with a significant portion achieving the maximum scores of 3 in the TIMI and TMP evaluations. There were no notable differences in the incidence of in-hospital MACE (Table 3).

**Table 3:** Pre- and post-intervention TIMI, TMP score, and ST-segment normalization

Characteristics	With TA (*n* = 68)		Without TA (*n* = 24)	
	Before	After	Before	After
TIMI score	0.9 ± .3	3.0 ± 0.2	1.1 ± 1.4	2.6 ± 0.9
	*p* < 0.001		*p* < 0.001	
TMP score	0.3 ± 0.7	2.9 ± 0.3	0.2 ± 0.6	2.0 ± 0.8
	*p* < 0.001		*p* < 0.001	
ST-segment normalization	59 (86.8)		6 (26.1)	
	*p* < 0.001			

Patients treated with TA have a significantly higher incidence of chest pain relief, ST-segment normalization, TIMI score of 3 achievement, and optimal reperfusion post-PCI, with p value < 0.001 (Tables 4, 5, 6).

**Table 4:** Post-procedure TIMI, TMP, complications, and major adverse cardiovascular events during hospitalization

Characteristics	Total (*n* = 92)	With TA (*n* = 68)	Without TA (*n* = 24)	*p*
	*n*(%)	*n*(%)	*n*(%)	
*TIMI, TMP post-procedure*				
TIMI of 3 after intervention	83 (91.2)	65 (95.6)	18 (78.3)	< 0.05^*^
TMP of 3 after intervention	65 (71.4)	61 (89.7)	4 (17.4)	< 0.001
*In-hospital complications*				
Arrhythmia	5 (5.4)	2 (2.9)	3 (12.5)	0.109^*^
Pneumonia	8 (8.7)	5 (7.4)	3 (12.5)	0.399^*^
Kidney failure	4 (4.3)	1 (1.5)	3 (12.5)	0.05^*^
Duration of hospitalization	5.0	5.0	5.0 (3.8–9.3)	0.699^*^
*In-hospital MACE*				
Death	4 (4.3%)	3 (4.4%)	1 (4.2%)	0.23
Stroke	4 (4.3%)	2 (2.9%)	2 (8.3%)	0.12
Recurrent acute myocardial infarction	0	0	0	NA
Thrombosis in stent	0	0	0	NA

^*^Fisher test

**Table 5:** The impact of TA on symptoms relief and ST-segment normalization post-PCI

Characteristics	No chest pain		OR (95% CI)	*p*	ST-segment back to normal		OR (95% CI)	*p*
	Yes (*n* %)	No (*n* %)			Yes (*n* %)	No (*n* %)		
With TA	59 (86.8)	9 (13.2)	8.52 (2.96–26.24)	< 0.001	59 (86.8)	9 (13.2)	18.57 (6.12–64.40)	< 0.001
Without TA	10 (43.5)	13 (56.5)			6 (26.1)	17 (73.9)

**Table 6:** The impact of TA on achieving TMP score of 3 and optimal reperfusion Post-PCI

Characteristics	TMP = 3		OR (95% CI)	*p*	Optimal reperfusion		OR (95% CI)	*p*
	Yes (n %)	No (n %)			Yes (n %)	No (n %)		
With TA	61 (89.7)	7 (10.3)	41.39 (12.0–179.09	< 0.001	53 (77.9)	15 (22.1)	77.73 (14.51–1449,77	< 0.001
Without TA	4 (17.4)	19 (82.6)			1 (4.3)	22 (95.7)		

**Fig. 3:**
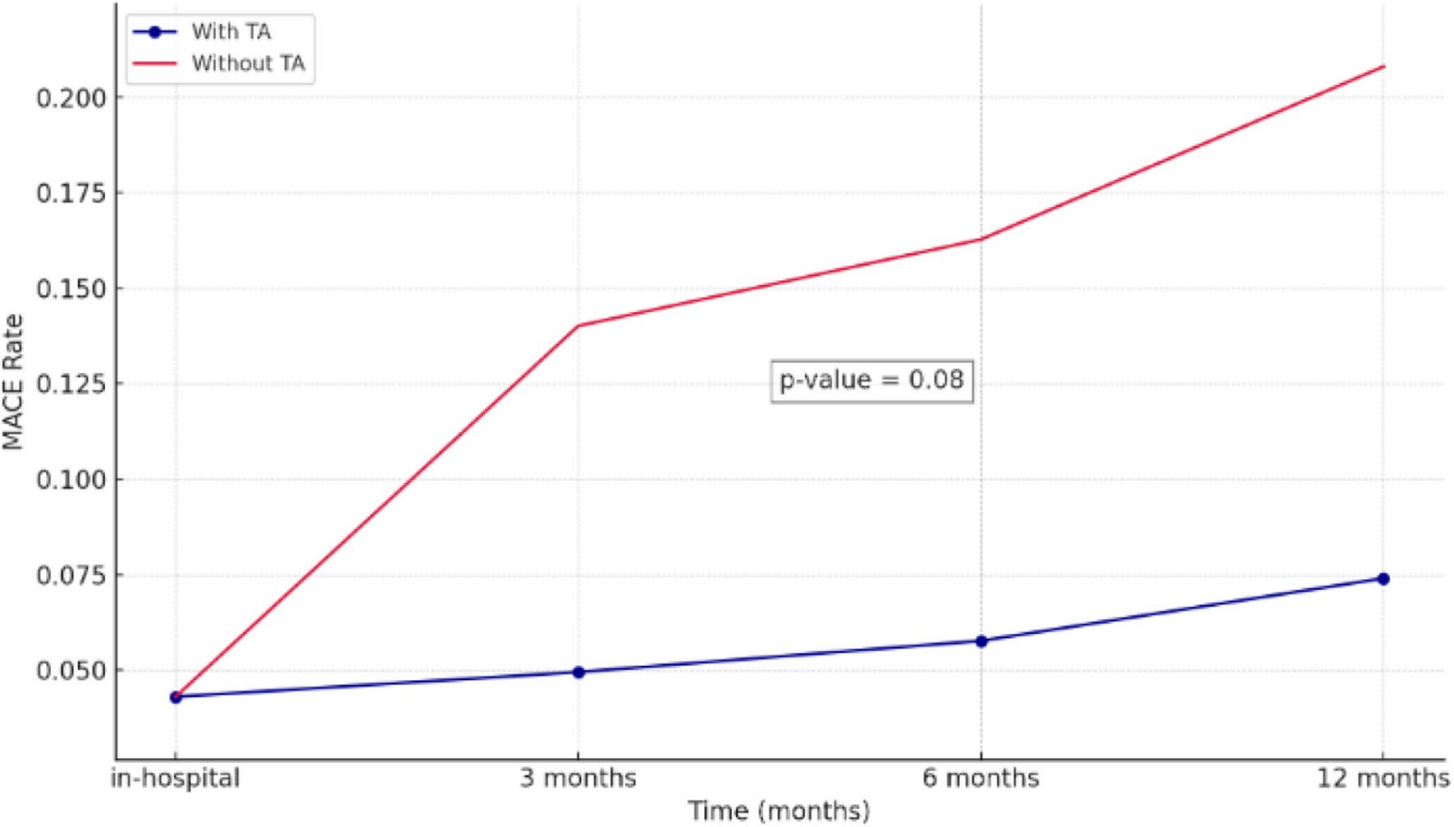
MACE comparison between TA and non-TA in STEM

For MACE outcomes, patients who received TA demonstrated a trend toward a lower incidence of MACE at both 30 days and 12 months after undergoing PCI, although the difference did not achieve statistical significance (Tables 7, S3 and Fig. 3).

**Table 7:** Major adverse cardiovascular events at 30 days and 12 months post-PCI

Characteristics	MACE 30 days		OR (95% CI)	*p*	MACE after 12 months		OR (95% CI)	*p*
	No n (%)	Yes n (%)			No n (%)	Yes n (%)		
With TA	65 (95.6)	3 (4.4)	0.32 (0.06–1.86)	0.186	63 (92.6)	5 (7.4)	0.30 (0.08–1.19)	0.08
Without TA	21 (87.5)	3 (12.5)			19 (79.2)	5 (20.8)

## Discussion

TA remains a controversial topic with mixed results from clinical trials. While smaller studies and meta-analyses have suggested that TA during primary PCI may reduce major adverse cardiovascular events (MACE) [12], more extensive randomized trials have not consistently confirmed these benefits. The REMEDIA (randomized evaluation of the effect of mechanical reduction of distal embolization by thrombus aspiration in primary and rescue angioplasty) trial in 2005 was among the first to show that TA improved reperfusion grades compared to standard PCI (46.0% vs. 24.5%, *p* < 0.001) [13]. The TAPAS (thrombus aspiration during percutaneous coronary intervention in acute myocardial infarction) trial in 2008 also found that TA led to better reperfusion and clinical outcomes than conventional PCI with a relatively low rate of adverse events at 30 days [14]. However, the following trials, TASTE (The Thrombus Aspiration in ST-Elevation Myocardial Infarction in Scandinavia) in 2013 and TOTAL (Trial of Routine Aspiration Thrombectomy with PCI versus PCI Alone in Patients with STEMI) in 2015 failed to demonstrate a reduction in cardiovascular events and death in the routine thrombus aspiration group [15, 16]. The presence of conflicting evidence has resulted in a Class III recommendation against the routine use of TA therapy in STEMI interventions in both the European Guidelines of 2017 and the ACC/AHA guidelines [9, 17].

Our study’s demographics and admission comorbidities align closely with those in major trials like TOTAL, TASTE, and CHEETAH. For example, the TOTAL trial reported a mean age of 61.0 years with 76.8% male participants, comparable to our cohort. However, our study showed higher rates of hypertension (56.5% vs. 50.3%) and diabetes mellitus (30.4% vs. 18.3%) [16]. Similarly, the TASTE trial reported lower rates of diabetes (13.3%) and hypertension (54.1%) [15]. These differences likely reflect the distinct demographic compositions in these RCTs, which predominantly included White and Hispanic patients, with minimal representation of Asians. It is important to note that research suggests Asian populations may have distinct characteristics and responses to myocardial infarction, including higher comorbidity rates and poorer outcomes in acute coronary syndrome (ACS)  [18,19]. Additionally, Asian populations often have lower mean BMI but higher rates of diabetes and metabolic syndrome compared to Western populations  [18, 20]. This underscores the necessity for region-specific data to inform clinical practice and bridge the knowledge gap regarding TA in PCI for STEMI patients in Vietnam.

Our research demonstrated favorable outcomes among STEMI patients with a high thrombus burden who underwent primary PPCI with TA, showing a significantly higher rate of achieving a TIMI flow score of 3 and a TMP grade of 3, as well as a greater frequency of ST-segment normalization compared to those who did not receive TA treatment (*p* < 0.001). These findings align with Ehab Mohamed Elfekky’s study in Egypt, which showed that TA reduced in-hospital mortality and improved TIMI flow grades, myocardial blush grade (MBG), ST-segment resolution, and left ventricular (LV) systolic function  [21]. Taku Inohara’s analysis utilizing the nationwide J-PCI registry also highlighted that TA was associated with a higher success rate of PCI procedures without significantly impacting in-hospital death among patients with STEMI, with an adjusted odds ratio (aOR) of 1.02 (95% CI, 0.94–1.12)  [22].

Regarding adverse outcomes, our study indicates a trend toward reduced MACE during a 12-month follow-up period in patients treated with TA, although this difference did not reach statistical significance. Similarly, Elfekky’s study showed that TA contributed to lower in-hospital mortality and a decreased prevalence of 30-day MACE  [21], while the TOTAL trial presented a trend toward a reduction in cardiovascular mortality at 30 days after TA (HR: 0.78, *p* = 0.06) [16]. Although some clinical trials have raised concerns about the routine use of TA due to a potentially increased risk of stroke within 30 days and at one year [16, 23], our study found no evidence of increased stroke events associated with TA in PPCI. These findings provide unique insights by focusing on a Vietnamese cohort, offering data that may better inform local clinical practices, especially in high-risk populations.

In general, our study collectively advocates for an appreciation of the TA’s role in managing STEMI patients, highlighting its potential to confer significant clinical benefits in carefully selected cases. For patients with high thrombus burdens, TA can serve as a valuable complement to conventional PCI. This approach can enhance cardiac outcomes by improving symptoms and myocardial perfusion post-intervention, all while not significantly elevating the risk of adverse events.

It is also essential to recognize the importance of a comprehensive approach, including lifestyle modifications in managing cardiovascular disease. Recent evidence highlights the role of the E(e)SEEDi lifestyle, which promotes sleep, emotion, exercise, and diet as key factors that significantly enhance patient outcomes [24]. This approach has been shown to improve prognosis, reduce recurrent events, and contribute to long-term cardiac health. Evidence suggests that adhering to a healthy lifestyle can lead to an 8–10% reduction in revascularization events within three years following PCI [25]. These findings reinforce the value of integrating lifestyle modifications alongside interventional procedures to potentially improve long-term outcomes for PCI patients. Future research should investigate the synergistic benefits of combining interventional treatments such as thrombus aspiration with structured lifestyle modifications, particularly in low- and middle-income countries where access to advanced treatments may be constrained.

### Limitation

This study has several limitations that should be considered. Firstly, the study’s findings are limited by the small sample size and the single-center, observational design, which may introduce selection bias and limit the generalizability of our results. Secondly, the relatively short follow-up period may not fully capture the long-term outcomes of the interventions. Furthermore, many outcomes were predominantly assessed through angiographic and electrocardiographic metrics, which, while widely used, offer only a limited view of the physiological recovery in myocardial perfusion.

Due to the current situation at our institute, we could not employ advanced imaging techniques such as positron emission tomography (PET) or magnetic resonance imaging (MRI) to assess myocardial perfusion, which limited the precision of our evaluation of perfusion improvements following thrombus aspiration. Additionally, the use of manual aspiration devices in our protocol, which may be less effective than advanced mechanical devices, could result in incomplete aspiration and suboptimal outcomes. In Vietnam, a middle-/low-income country, the high cost of mechanical devices (e.g., $4,000 to $5,000 for Penumbra versus $500 for Diver CE) significantly restricts their availability. Given these financial constraints, many patients cannot afford more expensive treatments, particularly as PCI is already costly. Further studies are needed to compare the outcomes of manual versus mechanical aspiration devices, which could provide critical insights for treatment decisions, especially in resource-limited settings.

The limitation of resources for diagnosis and treatment is not uncommon in low- and middle-income countries like Vietnam. We hope our study offers valuable evidence for physicians in similarly constrained environments, aiding in the optimization of treatment with limited resources and ultimately improving patient outcomes, despite the numerous aforementioned limitations.

## Conclusion

TA during primary percutaneous coronary intervention for patients with STEMI has shown the potential to enhance clinical outcomes. Benefits include the alleviation of chest pain, improved blood flow, and better myocardial perfusion without an increase in major adverse cardiovascular events at both 30-day and 12-month post-procedure follow-ups. Given these findings, TA may be a viable option for selected STEMI Vietnamese patients who have a significant thrombus burden.

## Data Availability

The datasets are available from the corresponding author upon reasonable request.

## Abbreviations

ACS: Acute coronary syndrome
ADP: Adenosine diphosphate
ACEI: Angiotensin-converting enzyme
aOR: Adjusted OR
ARB: Angiotensin receptor blocker
bpm: Beat per minute
ECG: Electrocardiographic
J-PCI: Japanese PCI registry
LMWH: Low molecular weight heparin
LV: Left ventricle
MACE: Major adverse cardiovascular events
MBG: Myocardial blush grade
MI: Myocardial infarction
PCI: Percutaneous coronary intervention
STEMI: ST-elevation myocardial infarction
TIMI: Thrombolysis in myocardial infarction
TMP: TIMI myocardial perfusion
